# Association of Integrated Mental Health Services with Physical Health Quality Among VA Primary Care Patients

**DOI:** 10.1007/s11606-021-07287-2

**Published:** 2022-02-09

**Authors:** Lucinda B. Leung, Lisa V. Rubenstein, Erin Jaske, Leslie Taylor, Edward P. Post, Karin M. Nelson, Ann-Marie Rosland

**Affiliations:** 1 Center for the Study of Healthcare Innovation, Implementation, & Policy, VA Greater Los Angeles Healthcare System, 11301 Wilshire Blvd (111G), Los Angeles, CA 90073 USA; 2 Division of General Internal Medicine and Health Services Research, UCLA David Geffen School of Medicine, Los Angeles, CA USA; 3 Department of Health Policy & Management, UCLA Fielding School of Public Health, Los Angeles, CA USA; 4 RAND Corporation, Santa Monica, CA USA; 5 VA Puget Sound Health Care System, Seattle, WA USA; 6 VA Ann Arbor, Center for Clinical Management Research, Ann Arbor, MI USA; 7 Department of Medicine, University of Michigan Medical School, Ann Arbor, MI USA; 8 Department of Medicine, University of Washington Medical School, Seattle, WA USA; 9 VA Center for Health Equity Research and Promotion, VA Pittsburgh Healthcare System, Pittsburgh, PA USA; 10 Department of Internal Medicine, University of Pittsburgh School of Medicine, Pittsburgh, PA USA

**Keywords:** Health services, Mental health, Diabetes, Hypertension, Veterans

## Abstract

**Background:**

Integrated care for comorbid depression and chronic medical disease improved physical and mental health outcomes in randomized controlled trials. The Veterans Health Administration (VA) implemented Primary Care–Mental Health Integration (PC-MHI) across all primary care clinics nationally to increase access to mental/behavioral health treatment, alongside physical health management.

**Objective:**

To examine whether widespread, pragmatic PC-MHI implementation was associated with improved care quality for chronic medical diseases.

**Design, Setting, and Participants:**

This retrospective cohort study included 828,050 primary care patients with at least one quality metric among 396 VA clinics providing PC-MHI services between October 2013 and September 2016.

**Main Measure(s):**

For outcome measures, chart abstractors rated whether diabetes and cardiovascular quality metrics were met for patients at each clinic as part of VA’s established quality reporting program. The explanatory variable was the proportion of primary care patients seen by integrated mental health specialists in each clinic annually. Multilevel logistic regression models examined associations between clinic PC-MHI proportion and patient-level quality metrics, adjusting for regional, patient, and time-level effects and clinic and patient characteristics.

**Key Results:**

Median proportion of patients seen in PC-MHI per clinic was 6.4% (IQR=4.7–8.7%). Nineteen percent of patients with diabetes had poor glycemic control (hemoglobin A1c >9%). Five percent had severely elevated blood pressure (>160/100 mmHg). Each two-fold increase in clinic PC-MHI proportion was associated with 2% lower adjusted odds of poor glycemic control (95% CI=0.96–0.99; *p*=0.046) in diabetes. While there was no association with quality for patients diagnosed with hypertension, patients without diagnosed hypertension had 5% (CI=0.92–0.99; *p*=0.046) lower adjusted odds of having elevated blood pressures.

**Conclusions and Relevance:**

Primary care clinics where integrated mental health care reached a greater proportion of patients achieved modest albeit statistically significant gains in key chronic care quality metrics, providing optimism about the expected effects of large-scale PC-MHI implementation on physical health.

**Supplementary Information:**

The online version contains supplementary material available at 10.1007/s11606-021-07287-2.

## INTRODUCTION

More than half of US adults have two or more chronic diseases, oftentimes a mix of physical and mental health conditions (comorbidity).^1^ For example, more than half of patients with depression have been documented having concurrent medical disease.^2^ Having both physical and mental health comorbidities is associated with significant negative impacts on condition management and health. Mental health comorbidities have been associated with approximately 10 to 20 years of premature mortality, mostly due to inadequately controlled physical health problems.^3^ Comorbidity is also costly to health systems. Medicare patients with comorbidity have 65% higher costs compared to those without comorbidity.^4^ Veterans who are at high risk for hospitalization in VA have also been found to heavily use both mental health and primary care services.^5^ As a result, many health systems strive to provide integrated mental health care modeled after evidence-based collaborative care which embeds mental health case managers and consulting psychiatrists in primary care settings.^6^

Growing evidence suggests that integrated mental health care can lead to better control of chronic physical health conditions, such as diabetes and cardiovascular disease.^7,8^ Few studies have been able to quantify the effects of integrated mental health care on physical health outcomes in pragmatic settings.^9^ Among fourteen clinics in one health system (*n*=218 patients), a randomized controlled trial (RCT) of collaborative care management for depression and diabetes reported significant reductions in hemoglobin A1c (HbA1c) (as compared to controls, 0.58% difference, over 12 months), and improvements in depression symptoms, cholesterol, blood pressure, and quality of life.^10^ Subsequently, the COMPASS trial replicated these findings when diabetes-depression collaborative care was implemented among 172 clinics in eight states (*n*=3609 patients).^11^ Despite evidence that integrated mental health care can be effective for managing comorbid chronic physical conditions, it is unclear whether positive patient outcomes extend to pragmatically (and variably) implemented integrated care models, i.e., outside of small patient populations and controlled clinical trial settings.^12^

Since 2008, the Veterans Health Administration (VA) has integrated mental health services into all primary care practices nationwide through its Primary Care–Mental Health Integration (PC-MHI) program, which is based on established collaborative care models.^13^ In 2010, the VA began implementing a multidisciplinary patient-centered medical home model called Patient Aligned Care Teams (PACT).^14^ While care model implementation can vary from site-to-site,^15^PC-MHI generally increased behavioral health staff and resources to support primary care teams in treating mental health conditions and addressing health behaviors including sleep, stress, and self-management of chronic physical health conditions. PC-MHI implementation has been found to increase patient use of outpatient primary and mental health care, and reduce non-mental health specialty care use, hospitalizations, and total costs of care among VA primary care patients.^16^ It is unknown whether these changes in utilization were related to PC-MHI-associated improvements in chronic physical health management. While one of the original goals of PC-MHI implementation was to improve mental health care access, it is possible that greater care integration led to positive indirect effects on the physical health of primary care patients. The widespread, successful implementation of PC-MHI across VA nationally offers an opportunity to examine effects of integrated mental health care on multiple chronic disease process and patient outcomes, to our knowledge, for the first time.

In this study, we examined, over 3 years, how the proportion of clinic patients seen by integrated mental health specialists was associated with patients’ diabetes and hypertension quality metrics in all VA primary care clinics mandated to have on-site PC-MHI programs full-time (i.e., serving 5,000+ patients). We hypothesized that patients assigned to primary care clinics with higher use of integrated mental health care would have higher odds of meeting chronic physical health condition control metrics (e.g., glycemic and blood pressure control), and secondarily, process metrics (e.g., diabetic retinopathy screening).

## METHODS

### Study Design and Cohort

This is a retrospective repeated cross-sectional study of 828,050 primary care patients whose health records were randomly sampled by VA’s quality monitoring program on October 1, 2013, to September 30, 2016. The VA External Peer Review Program^17^ reviews an independent probability sample of patient electronic health records across all VA clinics nationally. All patients sampled had a VA visit during the previous 13–24 months, and a VA primary care visit during the 12 months prior to and during the month preceding sampling. Patients were assigned to one of 396 VA primary care clinics (153 hospital-based, 243 community-based) mandated to offer on-site integrated mental health services. Cohorts were assembled for each of the following disease conditions: diabetes (*n*=135,819), hypertension (*n*=249,121), no diagnosed hypertension (*n*=166,000), cardiovascular disease (*n*=63,627).

### Measures

The explanatory variable (clinic-level) approximated the reach of integrated PC-MHI services within each primary care clinic.^16^ It was calculated as the proportion of assigned primary care patients who saw a PC-MHI specialist in each clinic per study year. Clinic PC-MHI proportion is an existing performance metric obtained from the VA Support Service Center (VSSC). Because PC-MHI programs were simultaneously implemented across all VA clinics nationwide, it is challenging to identify non-PC-MHI clinics as comparators for evaluation. PC-MHI proportion, which varies by clinic over time, has therefore been used in research studies to estimate the level of mental health integration within primary care clinics during each year of outcome measurement.^13,18,19^ For regression models, we performed log base 2 transformation to normalize data distribution of this variable.

Primary outcomes (patient-level) were measures of diabetes and hypertension control over 12 months, mostly analogous to Healthcare Effectiveness Data and Information Set (HEDIS) measures. For each patient, appropriate glycemic control was defined as HbA1c ≤ 9, versus >9 (or not done). Two metrics for appropriate hypertension control rated whether patients’ last blood pressure was <140/90, versus <160/100 mmHg (or not done). Secondary process outcomes related to chronic disease care included (1) whether patients with diabetes and/or cardiovascular disease had low-density lipoprotein (LDL) cholesterol <100 mg/dL or were taking moderate-intensity statin medications (data only available from fiscal years 2014–2015), and (2) whether patients with diabetes obtained timely retinal screening exams, annually (if retinopathy) or every 2 years (if no retinopathy).

Study covariates included clinic and patient-level variables chosen a priori from hypothesized associations with both care quality and mental health use in primary care. Patient age, gender, race-ethnicity, marital status, VA means test (which determines level of copayment charged for VA services), service-connected disability (which helps determine eligibility and payment for specific VA services), and distance from patient home to assigned primary care site were obtained from VA administrative data. We included each clinic’s PACT Implementation Progress Index (PI^2^), which measures degree of implementation of core PACT components (e.g., access, care coordination).^20^ PI^2^ has been found to be associated with chronic condition care quality,^21^ but not PC-MHI proportion.^16^ We also included clinic type (hospital- versus community-based) and location (rural versus nonrural). Clinic size was represented by the number of primary care patients assigned to that site at the end of each year.

We included patient mental health diagnoses (i.e., depression, anxiety, posttraumatic stress disorder, alcohol and substance use disorders [SUD], schizophrenia, bipolar disorder), defined by at least two occurrences of International Classification of Diseases (ICD)-9 and -10 diagnostic codes found in outpatient (at least one for SUD only) or one in inpatient encounter claims for each FY. We also indicated homeless status for any occurrence of ICD coding. We included Gagne et al.’s comorbidity score,^22^ which is based on conditions included in the Charlson Index and the Elixhauser comorbidity system. The score was subdivided into three levels of severity for each patient in each year, based on the VA primary care population distribution.

### Analyses

We described patient and clinic characteristics and mean proportion of patients meeting each quality outcome measure. In separate multilevel logistic regression models for each outcome quality metric, we examined how clinic PC-MHI proportion predicted patient odds of meeting the quality metric, controlling for year, VA region, clinic, and patient characteristics. Time (year) and regional fixed effects were included to account for secular trends and time-invariant regional effects. We also included patient random effects, because of presence of multiple non-independent observations per patient over 3 years. We accounted for multiple testing for all outcomes using the Benjamini-Hochberg Procedure.^23^

In sensitivity analyses, we restricted models to only patients with comorbid physical and mental health diagnoses, hypothesizing there would be greater impact on physical care quality among patients with diagnosed mental health needs. For all models, we determined significance using a two-tailed*α* of 0.05 and analyzed data in SAS version 9.4 and Stata version 14.

This study was conducted as part of an ongoing VA Office of Primary Care quality improvement effort to understand PC-MHI’s impact on care access and quality.^13,18,19^ It was designated as exempt from Institutional Review Board review.

### RESULTS

Among 828,050 unique VA primary care patients from 396 clinics with available quality monitoring data in FY2014 to FY2016 (*n*=1,130,371 person-years), 715,634 (86.4%) were men, 595,020 (71.9%) white, with a mean (standard deviation [SD]) baseline age of 63.7 (15.2) years (Table 1). Physical and mental health condition rates were high—205,853 (24.8%) had Gagne comorbidity scores classified in the highest category and 266,771 (32.2%) had a mental health diagnosis.

**Table 1:** Characteristics of Study Cohort Across 396 VA Primary Care Clinics, 2013–2016

	Number (%) (*n*=828,050 unique patients)
Age mean (SD) (years)	63.66 (15.2)
Female	112,416 (13.6)
Race/Ethnicity	
White, non-Hispanic	595,020 (71.9)
Black, non-Hispanic	144,354 (17.4)
Hispanic	53,304 (6.4)
Other	35,372 (4.3)
Marital status	
Single	106,056 (12.8)
Married	399,446 (48.2)
Divorced/Separated/Widowed	322,547 (39)
VA means test status	
Exempt	156,253 (18.9)
Any copay required	118,877 (14.4)
Testing not required, or missing	552,920 (66.8)
Service-connected disability rating	
0%	233,844 (33.5)
1–50%	158,659 (22.7)
51–100%	306,174 (43.8)
Homelessness documented in medical record	53,345 (6.4)
Gagne comorbidity score	
Low (bottom 25%)	123,554 (14.9)
Intermediate (middle 25–75%)	498,643 (60.2)
High (top 25%)	205,853 (24.9)
Mental health diagnoses	
Depression	266,771 (32.2)
Posttraumatic stress disorder	182,486 (22.0)
Substance use disorder	160,604 (19.4)
Anxiety disorder	145,963 (17.6)
Serious mental illness (e.g., schizophrenia, bipolar)	71,144 (8.6)
Average distance traveled to clinic (miles)	16.03 (16.8)
Primary chronic disease quality outcomes—physiologic control metrics	
*Diabetes related*	
Hemoglobin A1c >9 or not done (*n*=135,819)	26,599 (19.6)
Blood pressure >140/90, among patients with diabetes (*n*=135,819)	28,920 (21.3)
Blood pressure ≥ 160/100, or not done (*n*=135,819)	8,152 (5.3)
*Hypertension (HTN) related*	
Diagnosed HTN, blood pressure ≥ 160/100, or not done (*n*=249,121)	15,152 (6.1)
No HTN diagnosis, blood pressure >140/90 (*n*=166,000)	16,515 (9.9)
No HTN diagnosis, blood pressure ≥ 160/100, or not done (*n*=166,000)	5,069 (3.1)
Secondary chronic disease quality outcomes—process metrics	
No timely diabetes retinal exam (*n*=135,735)	14,000 (10.3)
LDL cholesterol >100 or no statin prescribed, among patients with diabetes (*n*=96,334)	12,942 (13.4)
LDL cholesterol >100 or no statin prescribed, among patients with cardiovascular disease (*n*=63,627)	7,316 (11.5)

Median clinic PC-MHI proportion (or percent of primary care patients seen by integrated mental health specialists) over the study period was 6.3% (range=0–36.4%). Clinics, on average, served 14,278 (SD=10,421) overall patients annually. Most clinics (75.4%) were in urban areas and were hospital-based (57.4%).

A total of 26,599 (19.6%) patients with diabetes had poor glycemic control (HbA1c >9% or not done) and 8,152 (5.3%) had severely elevated blood pressure (≥ 160/100 mmHg or not done). Among patients diagnosed with hypertension, 15,152 (6.1%) had severely elevated blood pressure. Among patients without diagnosed hypertension, 149,485 (90.1%) had blood pressure < 140/90 mmHg and 5,069 (3.1%) had severely elevated blood pressure > 160/100 mmHg or not done. A total of 121,734 (89.7%) patients with diabetes received timely retinal exams. Most patients with diabetes (83,392 [86.6%]) or cardiovascular disease (56,311 [88.5%]) had LDL cholesterol < 100 mg/dL or were prescribed moderate-dosed statins (Table 1).

In multivariate regression models, clinic PC-MHI proportion was significantly associated with glycemic control among patients with diabetes. Each two-fold increase in proportion of clinic patients seen by PC-MHI specialists was associated with lower odds of poor glycemic control (adjusted odds ratio [aOR]=0.98; 95% confidence interval [CI]=.96–.99) among the clinic’s patient population. However, clinic PC-MHI proportion was also associated with higher odds of not receiving timely retinal exams (aOR=1.04; CI=1.02–1.07) and not having LDL cholesterol <100 mg/dL or moderate-dosed statin prescription (aOR=1.03; CI=1.003–1.05) (Table 2). Appendix Fig. 1 shows the predicted percent of patients meeting quality metrics in clinics of varying PC-MHI proportions, holding all other covariates at the mean.

**Table 2:** Adjusted Odds of Meeting Chronic Disease Quality Metrics for Each Two-Fold Increase in the Proportion of Primary Care Clinic Patients Seen by PC-MHI Specialist

	Adjusted odds ratio (95% confidence interval)
Primary chronic disease quality outcomes—physiologic control metrics	
*Diabetes related*	
Hemoglobin A1c >9 or not done (*n*=135,819)	0.98 (0.96–0.99)*
Blood pressure >140/90, among patients with diabetes (*n*=135,819)	0.99 (0.97–1.00)
Blood pressure ≥ 160/100, or not done (*n*=135,819)	0.97 (0.94–1)
*Hypertension related*	
Diagnosed HTN, blood pressure ≥ 160/100, or not done (*n*=249,121)	0.99 (0.97–1.01)
No HTN diagnosis, blood pressure >140/90 (*n*=166,000)	0.98 (0.96–1.00)
No HTN diagnosis, blood pressure ≥ 160/100, or not done (*n*=166,000)	0.95 (0.92–0.99)*
Secondary chronic disease quality outcomes—process metrics	
No timely diabetes retinal exam (*n*=135,735)	1.04 (1.01–1.07)*
LDL cholesterol >100 or no statin prescribed, among patients with diabetes (*n*=96,334)	1.03 (1.01–1.06)*
LDL cholesterol >100 or no statin prescribed, among patients with cardiovascular disease (*n*=63,627)	1.04 (1.01–1.08)*

*LDL*, low-density lipoprotein. Odds ratios (and 95% confidence intervals) are derived from multilevel logistic regressions that controlled for clinic Primary Care–Mental Health Integration (PC-MHI) proportion, time (FY), region, patient characteristics (age, sex, race/ethnicity, marital status, service-connected disability, VA means testing, Gagne comorbidity score, homelessness status, distance between patients’ home addresses and their home clinics, and mental health diagnoses), and clinic characteristics (size, rurality, hospital- versus community-based, patient-centered medical home or Patient Aligned Care Teams implementation progress). *p<.05 after accounting for multiple testing using the Benjamini-Hochberg Procedure

While there was no observed association between clinic PC-MHI proportion and blood pressure control among veterans with diagnosed hypertension, we found lower odds of severely elevated blood pressure (aOR=0.95; CI=.92–.99) per two-fold increase in clinic PC-MHI proportion among patients *without* diagnosed hypertension. However, clinic PC-MHI proportion was also associated with higher odds of not having LDL cholesterol <100 mg/dL or moderate-dosed statin prescription (aOR=1.04; CI=1.01–1.07), among veterans with cardiovascular disease (Table 2).

Sensitivity analyses conducted by restricting cohort to primary care patients with mental health diagnoses yielded similar results for glycemic control among diabetes patients (Table 2, Appendix Table 3). In diabetes patients, each two-fold increase in proportion of clinic patients seen by PC-MHI specialists was associated with, on average, 4% lower odds of poor glycemic control (CI=.94–.99). No significant relationship was observed for other outcomes in this cohort.

## DISCUSSION

In this national study of 828,050 patients from nearly 400 primary care clinics that integrated mental health care, patients at clinics with higher proportions of patients seen by PC-MHI specialists had better glycemic and blood pressure control than those at clinics with lower proportions of patients seen by PC-MHI. Few studies have examined outcomes from real-world integrated mental health care programs in large health systems, wherein fidelity to collaborative care models as originally studied is often variable.^12,24,25^ This is the first study we are aware of that examines effects on physical health outcomes when integrated mental health care is widely and pragmatically implemented in a large health system, and that examines PC-MHI care across the full primary care population rather than among patients with specific physical health conditions. While effect sizes may seem modest, the population of patients with these chronic conditions is large, so even a small increase in integrated mental health use could lead to clinically significant increases in glycemic and blood pressure control.

Our findings show a significant relationship between the reach of a clinic’s primary care mental health integration program and physical health quality performance markers. Clinical trials studying collaborative care for patients with depression plus a specific physical health condition have consistently shown improvements in mental health outcomes (e.g., depression symptoms, mental health-related quality of life) but less consistently shown an impact on the concomitant physical health condition.^7^ Among these trials, collaborative care for depression and diabetes has been studied the most, and has the most documentation of impact on physical health outcomes (i.e., glycemic control).^10^ These studies, however, are RCTs engaging limited patient populations.^7,8^ This level of impact may not be achieved in the real-world setting, where significant variation in methods and quality of providing collaborative care^11^ and patient-centered medical home care in general varies widely.^20,26^ This study provides evidence that collaborative care at the full enrolled patient population level, as implemented across a variety of clinics and settings, can have positive impacts on physical health outcomes for two common physical health conditions.

While integrated mental health care does not typically directly address care of physical health conditions, there are multiple reasons it may have positive effects on diabetes and hypertension control. First, reduced mental health symptoms may make it easier for patients to manage their physical health.^27^ Additionally, better access to mental health care may help primary care clinicians to focus more effectively on managing physical illness.^28^ It is also possible that clinics with high use of integrated mental health care have underlying infrastructure or approaches that facilitate both PC-MHI and higher care quality for physical conditions.^29^ Our findings, however, showed slightly worse overall performance for diabetes exams and statin prescriptions as a clinic’s PC-MHI proportion increased, suggesting that the higher PC-MHI clinics may not have provided overall higher quality processes of care. The lack of PC-MHI-associated improvement in chronic disease care processes in the face of improvements in diabetes and hypertension control outcomes may suggest PC-MHI-associated improvements in patient self-management, a pathway that should be investigated in future research.

Research consistently demonstrates patients struggling with mental health conditions have worse physical health.^30–32^ VA patients with mental health diagnoses, however, have been shown to receive equivalent quality of care.^33^ Use of integrated mental health care in this study was also not associated with differences in quality improvements for diabetes and hypertension among patients with known mental health diagnoses than patients without. These findings suggest that patients with or without pre-existing mental health diagnoses benefit equally from PC-MHI, perhaps indicating that integrated care has broader effects on clinic performance than might be expected for an intervention directed at mental health alone. While VA’s PC-MHI care model was founded on evidence-based collaborative care to treat mental health conditions, as implemented in clinics, it has evolved to include additional activities for which little evidence on patient outcomes exists.^34,35^ Future research should investigate how PC-MHI might impact on physical condition care, including both through better recognition and management of patient distress and, possibly, improved patient-centered management of physical conditions by primary care providers and staff.

Our study has several limitations. First, the study examined an organizational-level explanatory variable, and not the direct effect of PC-MHI service exposure on individuals. While we were not able to include some pertinent organizational characteristics (i.e., primary care and/or mental health care staffing), we were able to control for others (e.g., rural location, clinic size) in our analyses. Second, our outcome data were derived by abstracting a sample of each clinic’s patient charts, albeit through an established representative method of monitoring VA care quality.^17^ Future research could compare results to newly available electronic quality measures collected for all VA patients.^36^ Third, our VA administrative data–based diagnosis information may be subject to coding differences because of the transition from ICD-9 to ICD-10 diagnostic codes, during which lower coding of certain mental health conditions (such as alcohol use disorders) has been documented.^37^ There is also likely variation in quality of similarly coded mental health services across providers. Finally, this study cannot account for how local versions of PC-MHI differed from each other, and thus cannot link specific program features (e.g., providing direct care for health behavior changes for chronic medical diseases) to results.

In summary, we found that, across a large health system with nationally implemented PC-MHI, patients cared for in primary care clinics where integrated mental health specialists reached a greater proportion of patients had significantly better diabetes and blood pressure control, than those in clinics with lower PC-MHI reach. Experiences of the VA—one of the largest programs to integrate mental health and primary care services to date—provide optimism about the expected effects of large-scale PC-MHI implementation on physical health outcomes. Future evaluations of health care system-wide implementation of integrated mental health care can further investigate the relationships between integrated primary and mental health care and patient physical health outcomes, including what specific clinic-level features of PC-MHI have the most impact.

## Supplementary information

ESM 1 (DOCX 148 kb)
