# Supplementary material for: Association of Integrated Mental Health Services with Physical Health Quality Among VA Primary Care Patients
Source: J Gen Intern Med. 2022 Feb 9;37(13):3331–7. doi: 10.1007/s11606-021-07287-2 (PMC9550947; doi:10.1007/s11606-021-07287-2)
Supplement: Supplementary file 1 — (DOCX 148 kb) [file 11606_2021_7287_MOESM1_ESM.docx]

**Appendix 1: Marginal predicted means (with 95% confidence intervals) of meeting chronic disease quality metrics at varying clinic PC-MHI proportions, based on significant multivariate regression models.**

**Diabetes Related**

**Hypertension (HTN) Related**


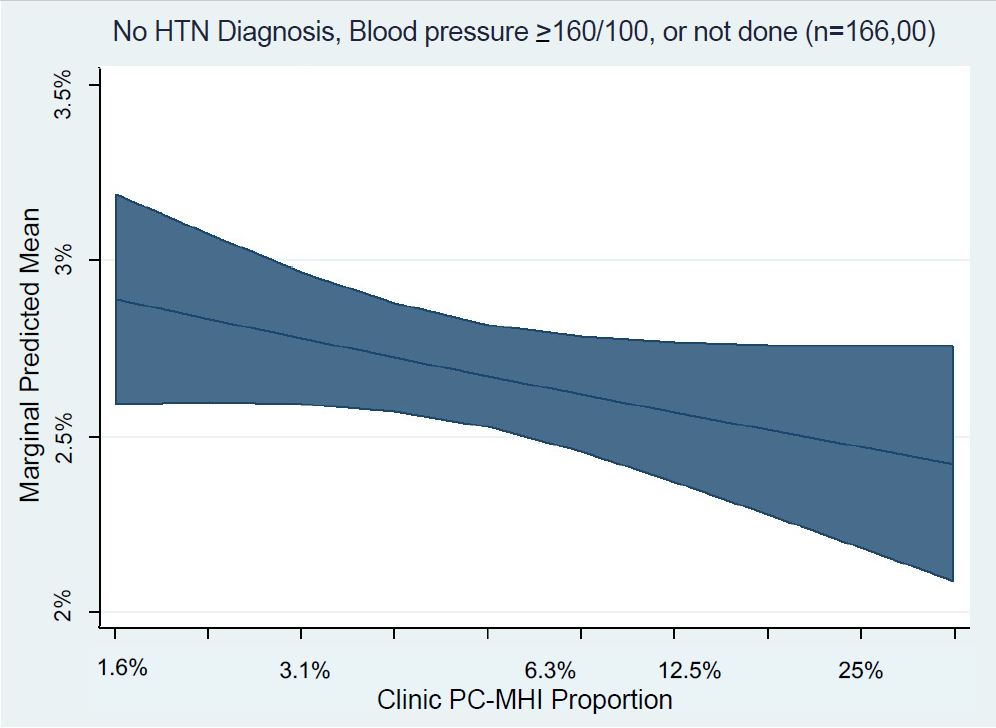

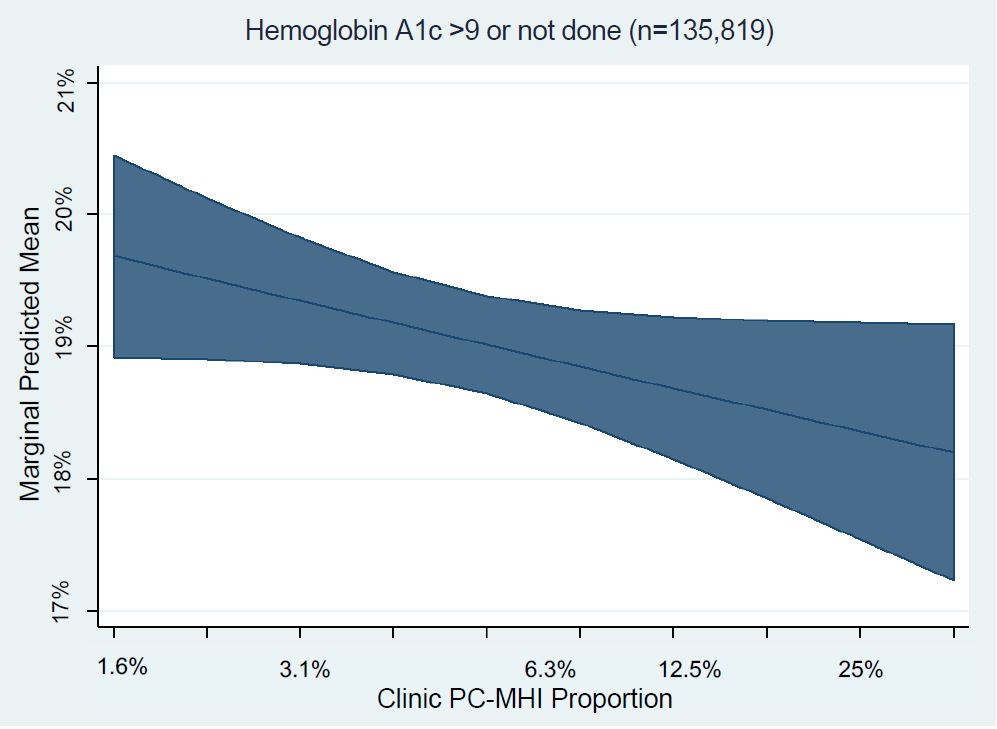


Marginal predicted means (and 95% confidence intervals) at varying clinic Primary Care–Mental Health Integration (PC-MHI) proportions are derived from multilevel logistic regressions that controlled for time (FY), region, patient characteristics (age, sex, race/ethnicity, marital status, service-connected disability, VA means testing, Gagne comorbidity score, homelessness status, distance between patients’ home addresses and their home clinics, and mental health diagnoses), and clinic characteristics (size, rurality, hospital- versus community-based, patient-centered medical home or Patient Aligned Care Teams implementation progress).

**Appendix 2: Adjusted odds of meeting chronic disease quality metrics for each two-fold increase in the proportion of primary care clinic patients seen by PC-MHI specialist (for subgroup of patients with diagnosed mental health conditions)**

|  | **Adjusted Odds Ratio (95% Confidence Interval)** | |  | |  |  |
| --- | --- | --- | --- | --- | --- | --- |
| **Primary Chronic Disease Quality Outcomes**  – Physiologic Control Metrics | | | | | | |
| *Diabetes Related* |  | |  | |  |  |
| Hemoglobin A1c >9 or not done (n=135,819) | 0.96 (0.94-0.99)* | |  |  |  |  |
| Blood pressure >140/90, among patients with diabetes (n=135,819) | 1.0 (1.0-1.01) | |  |  |  |  |
| Blood pressure ≥160/100, or not done (n=135,819) | 0.96 (0.92-1.01) | |  |  |  |  |
| *Hypertension Related* |  | | |  | |  |
| Diagnosed HTN, Blood pressure ≥160/100, or not done (n=249,121) | 0.99 (0.96-1.02) |  |  |  |  |  |
| No HTN Diagnosis, Blood pressure >140/90 (n=166,000) | 0.99 (0.97-1.01) |  |  |  |  |  |
| No HTN Diagnosis, Blood pressure ≥160/100, or not done (n=166,000) | 0.96 (0.92-1) |  |  |  |  |  |

Abbreviation: LDL= low-density lipoprotein; Odds ratios (and 95% confidence intervals) are derived from multilevel logistic regressions that controlled for clinic Primary Care–Mental Health Integration (PC-MHI) proportion, time (FY), region, patient characteristics (age, sex, race/ethnicity, marital status, service-connected disability, VA means testing, Gagne comorbidity score, homelessness status, distance between patients’ home addresses and their home clinics, and mental health diagnoses), and clinic characteristics (size, rurality, hospital- versus community-based, patient-centered medical home or Patient Aligned Care Teams implementation progress). *p<.05, †p<.01, ‡p<.001 after accounting for multiple testing using the Benjamini-Hochberg Procedure.
